# Genetic diversity and population structure of groundnut (*Arachis hypogaea* L.) accessions using phenotypic traits and SSR markers: implications for rust resistance breeding

**DOI:** 10.1007/s10722-020-01007-1

**Published:** 2020-09-05

**Authors:** Happy Daudi, Hussein Shimelis, Isack Mathew, Richard Oteng‐Frimpong, Chris Ojiewo, Rajeev K. Varshney

**Affiliations:** 1 African Centre for Crop Improvement, School of Agricultural, Earth and Environmental Sciences, College of Agriculture, Engineering and Science, University of KwaZulu-Natal, Pietermaritzburg, South Africa; 2 Tanzania Agricultural Research Institute-Naliendele, P.O. Box 509, Mtwara, Tanzania; 3 CSIR‐Savanna Agricultural Research Institute, Tamale, Ghana; 4 International Crops Research Institute for the Semi-Arid Tropics, Nairobi, Kenya; 5 International Crops Research Institute for the Semi-Arid Tropics, Hyderabad, India

**Keywords:** Agronomic traits, Gene diversity, Molecular variance, Polymorphism, Principal component analysis, Rust disease, SSR markers, Structure analysis, Tanzania

## Abstract

Groundnut (*Arachis hypogaea* L.) is a multi-purpose legume serving millions of farmers and their value chain actors globally. Use of old poor-performing cultivars contributes to low yields (< 1 t/ha) of groundnut in sub-Saharan Africa including Tanzania. The objectives of this study were to determine the extent of genetic variation among diverse groundnut collections using phenotypic traits and simple sequence repeat (SSR) markers to select distinct and complementary genotypes for breeding. One hundred and nineteen genotypes were evaluated under field conditions for agronomic traits and susceptibility to rust and leaf spot diseases. The study was conducted in two locations across two seasons. In addition, the 119 accessions were profiled with 13 selected SSR markers. Genotype and genotype by environment interaction effects were significant (*p* < 0.05) for days to flowering (DTF), late leaf spot score at 85 and 100 days after planting, pod yield (PDY), kernel yield (KY), hundred seed weight (HSW) and shelling percentage (SP). Principal components analysis revealed that plant stand, KY, SP, NPP (number of pods per plant), late leaf spot and rust disease scores accounted for the largest proportion of the total variation (71.9%) among the tested genotypes. Genotypes ICGV-SM 08587 and ICGV-SM 16579 had the most stable yields across the test environments. Moderate genetic variation was recorded with mean polymorphic information content of 0.34 and gene diversity of 0.63 using the SSR markers. The majority (74%) of genotypes showed high membership coefficients to their respective sub-populations, while 26% were admixtures after structure analysis. Much of the variation (69%) was found within populations due to genotypic differences. The present study identified genotypes ICGV-SM 06737, ICGV-SM 16575, ICG 12725 and ICGV-SM 16608 to be used for development of mapping population, which will be useful for groundnut improvement. This study provided a baseline information on characterization and selection of a large sample of groundnut genotypes in Tanzania for effective breeding and systematic conservation.

**Electronic supplementary material:**

The online version of this article (10.1007/s10722-020-01007-1) contains supplementary material, which is available to authorized users.

## Introduction

Cultivated groundnut (*Arachis hypogaea* L., AABB, 2*n* = 4x = 40) is an allotetraploid and a predominantly self-pollinating legume crop cultivated in most parts of the world. About 26.54 million hectares of groundnut is cultivated globally with an annual production of approximately 43.92 million tons of shelled grain (Upadhyaya et al. 2012; FAOSTAT 2014). Africa accounts for about 31.6% of the global production. However, most African countries do not meet their domestic demand for groundnuts. The sub-Saharan Africa (SSA) region has one of the lowest groundnut productivity levels (< 1 t/ha) in the world. FAOSTAT (2020) estimated monetary value of US$132 for importation of groundnut in Africa by 2020 to cover the shortfall due to low productivity in the region.

Groundnut productivity in Tanzania is < 1 t/ha compared to a mean yield of 2.5 t/ha elsewhere in Africa (FAOSTAT 2018). The low productivity is attributable to an array of abiotic and biotic constraints. The most notable biotic constraints include rust and late leaf spot diseases. Rust disease, caused by *Puccinia arachidis* Speg, is an important disease of cultivated groundnut that causes up to 57% yield loss (Mondal and Badigannavar 2015), while late leaf spot, *Cercosporidium personatum*, causes up to 50% yield loss (Branch and Culbreath 2013). Yield losses of up to 70% can be incurred when the two diseases occur simultaneously (Subrahmanyam et al. 1985; Khedikar et al. 2010). The damage symptoms associated with the occurrence of early rust attack include early pod maturity, reduced seed size, increased pod senescence, and decreased oil content (Mondal and Badigannavar 2015). Late leaf spot causes the plants to lose most or all the leaves, which significantly reduces photosynthetic efficiency (Branch and Culbreath 2013). Both rust and late leaf spot diseases can be controlled using a combination of methods such as cultural practices, biocontrol agents and host plant resistance (Mondal et al*.* 2014). Chemical control using fungicides requires repeated applications leading to concerns over high costs of production, environmental pollution, low quality of produce due to chemical residue, health of the farmer and the possibility of development of fungicide resistance in the pathogen. The use of chemicals to control rust and leaf spot is widespread but most of the smallholder farmers who depend on groundnut production in Tanzania cannot afford crop protection chemicals or may use sub-optimal rates leading to high yield losses due to the disease.

The incorporation of host resistance in susceptible groundnut genotypes is cost-effective and environmentally friendly disease control method and is widely regarded as the most sustainable and effective method. Improving rust and leaf spot resistance in groundnut will effectively improve productivity and reduce cost of production. Developing disease resistant cultivars depends on the availability and identification of sources of resistance. Resistance genes for rust and late leaf spot diseases have been identified in a wild relative of cultivated groundnut (*A. hypogaea*), elite inbred lines and commercial cultivars (Pande and Rao 2001; Fávero et al. 2015; Han et al. 2018). Improving resistance to rust in cultivated groundnut by introgressing resistance genes from wild *Arachis* species has been limited due to linkage drag associated with poor shelling, prominent reticulation and deep constriction in the pods (Dwivedi et al. 2003). There is a need to circumvent the unfavourable gene linkage by crossing divergent cultivated groundnut genotypes that harbour resistance genotypes. Hence, genetic variation among cultivated lines and landraces of groundnuts is more valuable for improving disease resistance because cultivated and elite inbred lines provide a readily available source of genes with potentially other farmer preferred traits.

Most groundnut genotypes grown in Tanzania are genetically diverse and unimproved landraces. These have not been tested for rust and leaf spot resistance, which could limit their use in breeding programs for developing rust or late leaf spot resistant cultivars with farmer-preferred traits. Therefore, screening the diverse germplasm maintained in Tanzania will contribute vital baseline information to facilitate selection of parental lines for cultivar development. The genetic pool initially acquired from ICRISAT-Malawi and maintained at Tanzania Agricultural Research Institute (TARI)-Naliendele station, forms part of important groundnut genetic resources in Tanzania.

Several studies that documented genetic variation in groundnut focused on using morphological traits (Ferguson et al. 2004; Bertioli et al. 2011; Nautiyal et al. 2011). Significant differences in growth habit, leaf number, number of pods, kernel weight and yield have been reported widely. This suggests that adequate morphological variation exists in groundnut for selection of genetically complementary and unique parents for breeding (Upadhyaya et al. 2009; Huang et al. 2015; Zhang et al. 2017). Despite significant morphological variation in groundnut, the limited genetic variability for enhanced yield and yield-related traits has been often cited as one of the reasons for little progress in genetic improvement of the crop (He et al. 2003). Morphological variations are largely influenced by environmental factors, which may affect the degree of trait heritability. Therefore, genotype screening should involve both phenotypic and molecular markers to elucidate the genetic potential of groundnut collections. In addition, there is a need to assess genetic variation and population structure of groundnut genetic resources using high throughput molecular markers.

Different molecular markers including amplified fragment length polymorphism (AFLP), restriction fragment length polymorphism (RFLP), random amplified polymorphic DNA (RAPD), single nucleotide polymorphisms (SNP) and microsatellites or simple sequence repeat (SSR) markers have been used in genetic variation studies on groundnut (Dwivedi et al. 2001; Mondal et al*.* 2008; Pandey et al. 2014; Vishwakarma et al. 2017). The choice of using each of the techniques is influenced by factors such as ease of application, genome coverage, costs, and automation compatibility. SSRs are highly preferred for their ability to detect high degrees of polymorphism, high reproducibility and abundant coverage of the genome (Pandey et al. 2012). In addition, SSR markers can be used for loci with multiple alleles and with co-dominant system (Gupta and Varshney 2000). Ren et al. (2014) and Wang et al. (2011) assessed genetic diversity and population structure in groundnut and found significant variation among Chinese cultivars and United States mini-core collections, respectively. Other studies have also reported the use of SSR markers in genetic analysis in groundnut (Mace et al*.* 2006; Mondal and Badigannavar 2010). However, the differences in the level of diversity across different germplasm collections and populations suggest that each population must be assessed in a target production environment for selection and systematic breeding program. Therefore, the objectives of this study were to determine the extent of genetic variation among germplasm from ICRISAT Malawi and landraces and varieties from Tanzania using phenotypic traits and SSR markers to select distinct and complementary genotypes for breeding. Data presented in the test populations provide useful information to deduce the population structure to devising a breeding strategy for enhanced yield and yield components and improved rust resistance by incorporating farmer-preferred traits in Tanzania.

## Materials and methods

### Plant materials

A total of 119 groundnut accessions (Table 1) were used in this study. The test accessions included ICRISAT’s breeding populations, landrace collections from different agro-ecologies in Tanzania and cultivated varieties (Table 1).

**Table 1:** Origin and description of groundnut acccessions used in the study

SN	Line	Pedigree	Origin*
1	ICGV-SM 16,554	(CG 7 X ICGV 02,194) F2-P9-P1-B1-B1-B1-B1	ICRISAT-Malawi
2	ICGV-SM 16555	(JL 24 X ICGV 02194)- F2-P2-P1-B1-B1-B1-B1	ICRISAT-Malawi
3	ICGV-SM 16556	(PENDO X ICGV 99557) F2-P4-P1-B1-B1-B1-B1	ICRISAT-Malawi
4	ICGV-SM 16557	(ICGV-SM 01711 X ICGV 02194) F2-P9-P1-B1-B1-B1-B1	ICRISAT-Malawi
5	ICGV-SM 16558	ICGV-SM 05701 X ICGV 02194) F2-P1-P1-B1-B1-B1-B1	ICRISAT-Malawi
6	ICGV-SM 16559	(ICGV-SM 01514 X ICGV 02194) F2-P7-P1-B1-B1-B1-B1	ICRISAT-Malawi
7	ICGV-SM 16560	(ICG 11426 X ICGV-SM 90704) F2-P14-P1-B1-B1-B1-B1	ICRISAT-Malawi
8	ICGV-SM 16561	(ICG 11426 X PENDO) F2-P11-P1-B1-B1-B1-B1	ICRISAT-Malawi
9	ICGV-SM 16562	(ICG 11426 X ICGV-SM 01721) F2-P21-P1-B1-B1-B1-B1	ICRISAT-Malawi
10	ICGV-SM 16563	(ICGV-SM 90704 X ICG 11426) F2-P3-P1-B1-B1-B1-B1	ICRISAT-Malawi
11	ICGV-SM 16564	PENDO X ICG 11426	ICRISAT-Malawi
12	ICGV-SM 16565	(ICGV-SM 01711 X ICG 11426) F2-P11-P1-B1-B1-B1-B1	ICRISAT-Malawi
13	ICGV-SM 16566	(ICGV-SM 99555 X ICG 11426) F2-P8-P1-B1-B1-B1-B1	ICRISAT-Malawi
14	ICGV-SM 16,567	(ICGV-SM 99557 X ICG 11426) F2-P14-P1-B1-B1-B1-B1	ICRISAT-Malawi
15	ICGV-SM 16,568	(ICGV-SM 05701X ICG 11426) F2-P11-P2-B2-B1-B1-B1	ICRISAT-Malawi
16	ICGV-SM 16569	(ICGV 01276 X CHALIMBANA) F2-P14-P1-B1-B1-B1-B1	ICRISAT-Malawi
17	ICGV-SM 16570	(ICGV 01276 X ICGV-SM 90704) F2-P15-P1-B1-B1-B1-B1	ICRISAT-Malawi
18	ICGV-SM 16571	(ICGV 01276 X ICGV-SM 90704) F2-P22-P1-B1-B1-B1-B1	ICRISAT-Malawi
19	ICGV-SM 16572	(ICGV 01276 X JL 24) F2-P3-P1-B1-B1-B1-B1	ICRISAT-Malawi
20	ICGV-SM 16573	CHALIMBANA X ICGV 01276	ICRISAT-Malawi
21	ICGV-SM 16574	ICGV-SM 90704 X ICGV 01276	ICRISAT-Malawi
22	ICGV-SM 16575	(CG 7 X ICGV 01276) F2-P8-P13-B1-B1-B1-B1	ICRISAT-Malawi
23	ICGV-SM 16576	(JL 24 X ICGV 01276) F2-P16-P1-B1-B1-B1-B1	ICRISAT-Malawi
24	ICGV-SM 16577	(PENDO X ICGV 01276) F2-P18-P1-B1-B1-B1-B1	ICRISAT-Malawi
25	ICGV-SM 16578	(ICGV-SM 01721 X ICGV 01276) F2-P6-P1-B1-B1-B1-B1	ICRISAT-Malawi
26	ICGV-SM 16,579	(ICGV-SM 99555 X ICGV 01276) F2-P4-P1-B1-B1-B1-B1	ICRISAT-Malawi
27	ICGV-SM 16580	(ICGV-SM 05701 X ICGV 01276) F2-P8-P1-B1-B1-B1-B1	ICRISAT-Malawi
28	ICGV-SM 16581	(ICGV-SM 01514 X ICGV 01276) F2-P1-P2-B1-B1-B1-B1	ICRISAT-Malawi
29	ICGV-SM 16582	ICGV 02286 X CHALIMBANA	ICRISAT-Malawi
30	ICGV-SM 16583	ICGV 02286 X ICGV-SM 90704	ICRISAT-Malawi
31	ICGV-SM 16584	(ICGV 02286 X CG 7) F2-P21-P1-B1-B1-B1-B1	ICRISAT-Malawi
32	ICGV-SM 16585	ICGV 02286 X ICGV-SM 05701	ICRISAT-Malawi
33	ICGV-SM 16586	(ICGV 02286 X ICGV-SM 05701) F2-P1-P3-B1-B1-B1-B1	ICRISAT-Malawi
34	ICGV-SM 16587	(ICGV 02286 X ICGV-SM 05701) F2-P1-P4-B1-B1-B1-B1	ICRISAT-Malawi
35	ICGV-SM 16588	ICGV 02286 X ICGV-SM 05701	ICRISAT-Malawi
36	ICGV-SM 16589	(ICGV 02286 X ICGV-SM 05701) F2-P1-P14-B1-B1-B1-B1	ICRISAT-Malawi
37	ICGV-SM 16590	ICGV 02286 X ICGV-SM 05701	ICRISAT-Malawi
38	ICGV-SM 16591	(ICGV 02286 X ICGV-SM 05701) F2-P1-P20-B1-B1-B1-B1	ICRISAT-Malawi
39	ICGV-SM 16592	(ICGV 02286 X ICGV-SM 05701) F2-P1-P24-B1-B1-B1-B1	ICRISAT-Malawi
40	ICGV-SM 16593	(ICGV 02286 X ICGV-SM 05701) F2-P1-P27-B1-B1-B1-B1	ICRISAT-Malawi
41	ICGV-SM 16,594	(ICGV 02286 X ICGV-SM 05701) F2-P1-P28-B1-B1-B1-B1	ICRISAT-Malawi
42	ICGV-SM 16595	(ICGV 02286 X ICGV-SM 05701) F2-P1-P29-B1-B1-B1-B1	ICRISAT-Malawi
43	ICGV-SM 16597	(ICGV 02286 X ICGV-SM 05701) F2-P1-P31-B1-B1-B1-B1	ICRISAT-Malawi
44	ICGV-SM 16598	(ICGV 02286 X ICGV-SM 05701) F2-P1-P39-B1-B1-B1-B1	ICRISAT-Malawi
45	ICGV-SM 16599	ICGV 02286 X ICGV-SM 05701	ICRISAT-Malawi
46	ICGV-SM 16600	(ICGV 02286 X ICGV-SM 05701) F2-P1-P41-B1-B1-B1-B1	ICRISAT-Malawi
47	ICGV-SM 16601	(ICGV 02286 X ICGV-SM 05701) F2-P1-P44-B1-B1-B1-B1	ICRISAT-Malawi
48	ICGV-SM 16602	(ICGV 02286 X ICGV-SM 05701) F2-P1-P49-B1-B1-B1-B1	ICRISAT-Malawi
49	ICGV-SM 16603	(ICGV 02286 X ICGV-SM 05701) F2-P1-P50-B1-B1-B1-B1	ICRISAT-Malawi
50	ICGV-SM 16604	(ICGV 02286 X ICGV-SM 05701) F2-P1-P53-B1-B1-B1-B1	ICRISAT-Malawi
51	ICGV-SM 16605	(ICGV 02286 X ICGV-SM 05701) F2-P1-P54-B1-B1-B1-B1	ICRISAT-Malawi
52	ICGV-SM 16606	ICGV 02286 X ICGV-SM 05701	ICRISAT-Malawi
53	ICGV-SM 16607	ICGV 02286 X ICGV-SM 05701	ICRISAT-Malawi
54	ICGV-SM 16608	(ICGV 02286 X ICGV-SM 05701) F2-P1-P257-B1-B1-B1-B1	ICRISAT-Malawi
55	ICGV-SM 16609	(ICGV 02286 X ICGV-SM 05701) F2-P1-P58-B1-B1-B1-B1	ICRISAT-Malawi
56	ICGV-SM 16610	ICGV 02286 X ICGV-SM 05701	ICRISAT-Malawi
57	ICGV-SM 16611	(ICGV 02286 X ICGV-SM 05701) F2-P1-P60-B1-B1-B1-B1	ICRISAT-Malawi
58	ICGV-SM 16612	(ICGV 02286 X ICGV-SM 05701) F2-P1-P62-B1-B1-B1-B1	ICRISAT-Malawi
59	ICGV-SM 16,613	(ICGV 02286 X ICGV-SM 05701) F2-P1-P64-B1-B1-B1-B1	ICRISAT-Malawi
60	ICGV-SM 16614	(ICGV 02286 X ICGV-SM 05701) F2-P1-P65-B1-B1-B1-B1	ICRISAT-Malawi
61	ICGV-SM 16615	(ICGV 02286 X ICGV-SM 05701) F2-P1-P67-B1-B1-B1-B1	ICRISAT-Malawi
62	ICGV-SM 16616	(ICGV 02286 X ICGV-SM 05701) F2-P1-P68-B1-B1-B1-B1	ICRISAT-Malawi
63	ICGV-SM 16617	(ICGV 02286 X ICGV-SM 01514) F2-P1-P2-B1-B1-B1-B1	ICRISAT-Malawi
64	ICGV-SM 16618	(ICGV 02286 X ICGV-SM 01514) F2-P1-P5-B1-B1-B1-B1	ICRISAT-Malawi
65	ICGV-SM 16619	(ICGV 02286 X ICGV-SM 01514) F2-P1-P6-B1-B1-B1-B1	ICRISAT-Malawi
66	ICGV 93542	ICGV 93542	ICRISAT-Malawi
67	ICGV-SM 15510	ICGV 93437 × ICGV 95342	ICRISAT-Malawi
68	ICGV-SM 15514	(ICGV 93437 × ICGV 95342) F2-P35-P6-B1-B1-B1-B1	ICRISAT-Malawi
69	ICGV-SM 15524	(ICGV 93437 × ICGV 95342) F2-P55-P53-B1-B1-B1-B1	ICRISAT-Malawi
70	ICGV-SM 15529	(ICGV 93437 × ICGV 95342) F2-P63-P41-B1-B1-B1-B1	ICRISAT-Malawi
71	ICGV-SM 15531	ICGV 95342 × ICGV 93437	ICRISAT-Malawi
72	ICGV-SM 15534	(ICGV 95342 × ICGV 93437) F2-P3-P23-B1-B1-B1-B1	ICRISAT-Malawi
73	ICGV-SM 15536	(ICGV 94114 × JL 24) F2-P51-P10-B1-B1-B1-B1	ICRISAT-Malawi
74	ICGV-SM 15537	(ICGV 94114 × JL 24) F2-P50-P19-B1-B1-B1-B1	ICRISAT-Malawi
75	ICGV-SM 15538	(ICGV 94114 × JL 24) F2-P50-P14-B1-B1-B1-B1	ICRISAT-Malawi
76	ICGV-SM 15542	(ICGV 94114 × JL 24) F2-P35-P13-B1-B1-B1-B1	ICRISAT-Malawi
77	ICGV-SM 15546	ICGV 94114 × JL 24	ICRISAT-Malawi
78	ICGV-SM 15548	(ICGV 94114 × JL 24) F2-P9-P21-B1-B1-B1-B1	ICRISAT-Malawi
79	ICGV-SM 15554	(JL 24 × ICGV 94114) F2-P134-P7-B1-B1-B1-B1	ICRISAT-Malawi
80	ICGV-SM 15556	(JL 24 × ICGV 94114) F2-P113-P1-B1-B1-B1-B1	ICRISAT-Malawi
81	ICGV-SM 15557	(JL 24 × ICGV 94114) F2-P102-P13-B1-B1-B1-B1	ICRISAT-Malawi
82	ICGV-SM 15558	(JL 24 × ICGV 94114) F2-P93-P11-B1-B1-B1-B1	ICRISAT-Malawi
83	ICGV-SM 15,559	(JL 24 × ICGV 94114) F2-P93-P4-B1-B1-B1-B1	ICRISAT-Malawi
84	ICGV-SM 15562	(JL 24 × ICGV 94114) F2-P65-P33-B1-B1-B1-B1	ICRISAT-Malawi
85	ICGV-SM 15564	(JL 24 × ICGV 94114) F2-P65-P22-B1-B1-B1-B1	ICRISAT-Malawi
86	ICGV-SM 15567	(JL 24 × ICGV 94114) F2-P27-P27-B1-B1-B1-B1	ICRISAT-Malawi
87	ICGV-SM 90704	(RG 1 × Manipintar) F2-P23-P59-P59-B1-B1-B13-B1	ICRISAT-Malawi
88	ICGV 94114	(J11 x CS 31) F2-B1-B1-B1-B1-B2-B1-B1-B2-B1	ICRISAT-Malawi
89	ICGV-SM 08578	ICGV 90082 X ICGV-SM 94581	ICRISAT-Malawi
90	ICGV-SM 08587	ICGV 90082 X ICGV 90092	ICRISAT-Malawi
91	ICGV-SM 08586	ICGV 90082 X ICGV 90092	ICRISAT-Malawi
92	CG 7	(USA 20 × TMV 10) F2-P3-B1-B1-B1-B1-B1B1-B1-B1	ICRISAT-Malawi/released variety
93	ICGV-SM 08581	ICGV 90082 X ICGV 90092	ICRISAT-Malawi
94	ICG 12725	ICG 12725	ICRISAT-Malawi
95	ICGV-SM 05570	ICGV 90103 X PC 223 K9	ICRISAT-Malawi
96	ICGV 94124	(ICGV 87314 × NCAC 343) F2-B2-B1-B1-B1	ICRISAT-Malawi
97	ICGV-SM 06718	ICGV 90103 X ICGV 92092	ICRISAT-Malawi
98	ICGV-SM 05611	ICGV 92092 X ICG 9991	ICRISAT-Malawi
99	ICGV-SM 05569	ICGV 90103 X ICGV 92092	ICRISAT-Malawi
100	ICGV-SM 08584	ICGV 90082 X ICGV 90092	ICRISAT-Malawi
101	ICGV-SM 06735	ICGV 9003 X ICGV 92092	ICRISAT-Malawi
102	ICGV 95342	[(ICG(FDRS)33 × ECZ1135) x (ICG (FDRS) x J11)] F2-F1-B1-B2-B2-B1-B1-B1-B1-B2-B1-B1-B1	ICRISAT-Malawi
103	ICGV-SM 05616	ICGV 90100 X JL 24	ICRISAT-Malawi
104	ICGV-SM 87,157	ICGV-SM 87,157	ICRISAT-Malawi
105	ICGV-SM 06711	ICGV 90103 X ICGV 92092	ICRISAT-Malawi
106	ICGV-SM 06737	ICGV 90103 X ICGV 9292	ICRISAT-Malawi
107	ICG 10879	ICG 10879	ICRISAT-Malawi
108	ICGV-SM 01514	(ICGV 93437 X ICGV-SM 93561)-ICGX-SM 95041/6/P15/P3	ICRISAT-Malawi
109	Masasi 09	ICGV-SM 87727 × ICGV-SM 83708	TARI-Naliendele/released variety
110	Pendo 98	ICGMS -33	TARI-Naliendele/released variety
111	Narinut 15	ICGV-SM 87727 × ICGV-SM 83708	TARI-Naliendele/released variety
112	Mangaka 09	ICGV 93437 × ICGV-SM 94586	TARI-Naliendele/released variety
113	Naliendele 09	ICGV-SM 93437 × ICGV-SM 94586	TARI-Naliendele/released variety
114	Nachingwea 09	ICGV-SM 90704 × ICGV-SM83708	TARI-Naliendele/released variety
115	Kanyomwa	Na	Landrace (Nanyumbu)
116	Local Dodoma	Na	Landrace (Dodoma)
117	Mamboleo	Na	Landrace (Dodoma)
118	Local Tandahimba	Na	Landrace (Tandahimba)
119	Ndulima	Na	Landrace (Nanyumbu)

*SN* serial number, *Na* not available, *ICRISAT* International Crops Research Institute for the Semi-Arid Tropics, *TARI* Tanzania Agricultural Research Institute

*names in parenthesis show collections areas in Tanzania

### Phenotyping

#### Site description

The 119 accessions were evaluated at two research sites of the Tanzania Agriculture Research Institute (TARI) namely Naliendele Agricultural Research Centre and Chambezi Experimental Station. The genotypes were screened for resistance to rust disease and late leaf spot during the 2018 and 2019 seasons. TARI-Naliendele (10.3539°S, 40.1682°E) is situated at an altitude of 135 m above sea level (masl). The mean monthly temperatures for TARI-Naliendele ranges between 24.3 °C in July and 27 °C in December while the mean annual rainfall is between 820 and 1245 mm with a unimodal rain distribution. A dry spell of one to two weeks often occurs at the end of January or at the beginning of February. The soils at TARI-Naliendele described as sandy loam with pH of 4.5. Chambezi Experimental Station (06.5167°S, 38.9167°E) is located at an altitude of 12 masl. The monthly temperatures at Chambezi vary between 24 °C in September and 30 °C in February. The site is characterized by a bi-modal rainfall pattern, commencing from October to December and April to June with expected dry spells from January to March. The annual rainfall ranges between 600 and 1000 mm, which is marked by high variation in amount and distribution. The soils at Chambezi were also sandy loam with a pH of 5.0.

### Experimental design and trial establishment

The experiment was conducted under field conditions over two seasons and laid out using an 8 × 15 alpha lattice design with two replications. Each genotype was planted on a plot consisting of two rows that were four metres long. The inter-row spacing was 50 cm with an intra-row spacing of 10 cm. The total plot size for each genotype was 4.0m^2^. The recommended practices for fertilizer application and weeding in Tanzania were followed (NARI 2001). The trials at Chambezi were established under natural rainfall and TARI-Naliendele under natural rainfall and supplemental sprinkler irrigation when required. These sites are hotspots for rust and late leaf spot diseases. Hence, the genotypes were evaluated under natural disease infection. A susceptible genotype, Pendo 98, was planted next to each plot serving as a disease spreader through maintaining effective inoculum source for test genotypes.

### Data collection

Data on yield and yield components were recorded during plant growth and at harvest maturity. The initial plant stand (IPS) was determined by counting the number of plants in each plot after germination. Days to 75% flowering (DTF) were recorded by counting the number of days from sowing to the time when 75% of the plot stand had reached flowering. Plant height (PH, expressed in cm) was measured from ten randomly sampled plants in each plot from the soil surface to the tip of main stem. The number of pods per plant (NPP) was recorded as the average number of pods from ten randomly sampled plants. Final plant stand (FPS) was recorded as the number of plants in each plot before harvesting. Pod yield (PDY) was measured by weighing the dried pods from each plot and was recorded in grams per plot. Shelling percentage (SP) for each genotype was calculated from a random sample of pods weighing 200 g, as the proportion of shelled seed weight to the total weight of the unshelled pods. Additionally, 100 seed weight (HSW, expressed in grams) for each genotype was recorded as an average weight of two samples of 100 randomly selected kernels per plot. Kernel yield (KY, expressed in t ha^−1^) was estimated as the product of pod yield per plot and shelling percentage and was converted to t ha^−1^ accordingly, using the plot size after adjusting for moisture content.

Rust severity was scored twice at 85 and 100 days after planting. The severity score at 85 days is represented as %RI85 while at 100 days it is designated as %RI100. Severity was scored using a scale of 1 (least affected) to 9 (most affected) (Das et al. 1999). Plants with no symptoms of infection were assigned a disease score of 1 (for 0% infection) while leaves with 1–5% infection were assigned a score of 2, 6–10% infection (score 3), 11–20% infection (score 4), 21–30% (score 5), 31–40% infection (score 6), 41–60% infection (score 7), 61–80% infection (score 8) and 81–100% infection (score 9) (Subbarao et al. 1990). Plants with a disease score of 1–3, 4–6 and 7–9 were considered to be resistant, moderately resistant and susceptible, respectively (Pande et al. 2002). In addition, late leaf spot reaction was assessed as a secondary trait. Late leaf spot disease often occurs simultaneously with rust disease. The screening procedure and scoring for late leaf spot was like the one used for rust disease.

### Genotyping

Seeds of the 119 groundnut accessions were sown under greenhouse conditions at TARI-Naliendele, Tanzania. Ten seeds per genotype were planted and allowed to establish for 20 days. Five healthy and randomly selected leaves were sampled per genotype for DNA extraction. The leaves were sun dried after collection and then packed in paper bags with silica gel before shipment to the Centre of Excellence in Genomics and Systems Biology, ICRISAT in India. The Cetyl-tetramethyl ammonium bromide (CTAB) procedure was followed during DNA extraction (Cuc et al. 2008).The DNA quality and quantity were checked on nanodrop and DNA concentration was normalized to ~ 10 ng/µl for further genotyping with the linked markers.

A total of 13 SSR markers were used in the study (Table 2). The markers used in this study were purposefully selected because of their suitability in discriminating groundnut genotypes for rust resistance. The markers showed high polymorphic information content and recommended for genetic analysis in groundnut. These were amplified using the polymerase chain reaction (PCR) following the procedures outlined by (Khedikar et al. 2010; Sujay et al. 2012). The PCR amplicons of the linked markers were separated as described in Varshney et al*.* (2009a).

**Table 2:** Names and sequence information of the 13 SSR markers used for genetic analysis

SN	Marker	Forward sequence	Reverse sequence	Reference
1	IPAHM103	GCATTCACCACCATAGTCCA	TCCTCTGACTTTCCTCCATCA	Cuc et al. (2008)
2	GM2301	GTAACCACAGCTGGCATGAAC	TCTTCAAGAACCCACCAACAC	Varshney et al. (2014)
3	TE 360	GGGATATGATGCCCATAGCTGA	TGCTGACTACTTGCAATGCC	Mondal et al. (2014)
4	TE 498	ATGACTTACATGTAGCAATTG	TGAAAGGAGTCAAAGGTCATG	Mondal et al. (2014)
5	PM 050	CAATTCATGATAGTATTTTATTGGACA	CTTTCTCCTCCCCAATTTGA	He et al. (2003)
6	PM179	CTGATGCATGTTTAGCACACTT	TGAGTTGTGACGGCTTGTGT	He et al. (2003)
7	pPGPseq-17F6	CGTCGGATTTATCTGCCAGT	AGTAGGGGCAAGGGTTGATG	Mace et al. (2006)
8	pPGPseq-16C6	TTGCTACTAAGCCGAAAATGAAG	CTTGAAATTAACACATATGCACACA	Mace et al. (2006)
9	pPGPseq-8E12	TCTGTTGAGAACCACCAGCA	GTGCTAGTTGCTTGACGCAC	Moretzsohn et al. (2005)
10	pPGPseq-10D4	ATCCCTGATTAGTGCAACGC	CGTAGGTGGTTTTAGGAGGG	Moretzsohn et al. (2005)
11	pPGPseq-12F7	TGTCGTTGTAAGACCTCGGA	TTGGTTTCCTTAAGGCTTCG	Moretzsohn et al. (2005)
12	pPGPseq-13A10	AACTCGCTTGTACCGGCTAA	AGGAATAATAACAATACCAACAGCA	Moretzsohn et al. (2005)
13	SSR_HO115759	TATCAACGCAACCTTTTGCAG	GACTTGTGTGGCTGAAACTTGA	Mondal et al. (2012)

A 10 μl PCR mix containing 15 mM of magnesium chloride, 2 μl dNTPs, 5u/ul Taq, 10 pm/ul primer, 10 × PCR buffer and 5.95MilliQ H_2_O was used for PCR amplification. The initial denaturation temperature was set at 94 °C with subsequent 10 rounds of denaturing at -1 °C. Annealing was conducted at 55 °C for 10 secs while the PCR substrates were set for at 72 °C for 20 s to allow for extension. Thereafter, the samples were visualized by fluorescence using the Genetic Analyser 3130xl and electrophoresis was conducted on an ABI 3013 automatic sequencer. Allele sizing of the electropherograms was carried out using GeneMapper V4 software and the fragment sizes were provided as Excel output.

### Phenotypic data analyses

The phenotypic data was subjected to analysis of variance (ANOVA) to test the effects of genotypes and locations and their interaction using the restricted maximum likelihood model (REML) procedure for alpha lattice designs in GenStat 18^th^ edition (Payne 2015). The means were separated by the Fischer’s unprotected least significant difference at 0.05. The correlations among the traits were based on the Pearson correlation coefficients conducted in R (R Core Team 2019). Multivariate analysis using the principal components was conducted using the Statistical Package for Social Science (SPSS) software version 24 (Kirkpatrick and Feeney 2012). The genotype and genotype × environment interaction (Singh et al. 2012) analysis was performed to test the effects of genotypes and environments, and their interaction. The effects of genotype, genotype × environment interaction were visualized graphically using the GGE biplot constructed in Genstat 18^th^ edition (Goedhart and Thissen 2010). The GGE biplots were based on the first two principal components (PC1 and PC2) after compressing multi-environment data into a single value (Yan et al. 2001). Two GGE biplots were constructed for visual assessments, one focused on the genotype differences while the other depicting the environmental variation.

### Genotypic data analyses

The major allele frequency, the number of effective alleles, heterozygosity and gene diversity were calculated using the simple allele frequency estimator while polymorphic information content values were estimated using the equation below (Botstein et al. 1980).

PIC = 1–Σ (pi^2^), where pi is the frequency of ith allele.

Hierarchical cluster analysis was conducted based on Ward minimum variance test using R statistical software (R Core Team 2019). The cluster patterns were visualized using factoextra package (Kasambara and Mundt 2017) in the R statistical software. The population structure was inferred using Structure 2.0 software (Falush et al. 2003). The optimal number of subpopulations (K) was identified based on maximum likelihood and delta K (△*K*) values (Evanno et al. 2005). The STRUCTURE program was run 10 times for each K value using the admixture model and correlated allele frequency, with 20,000 burn-in period and 10 000 Markov Chain Monte Carlo (MCMC) iterations during analysis. A repeat run with 50,000 burn in and 100,000 MCMC iterations was carried out to confirm the best K value.

Analysis of molecular variance (AMOVA) was conducted using PowerMarker software version 3.25 (Liu and Muse 2005) to partition genetic variation between and among populations. Significance of estimated variance components was based on 10,000 random permutations.

## Results

### Genetic variation among groundnut accessions

The ANOVA revealed that the 3-way interaction involving genotype, location and season had significant (*p* < 0.05) impact on IPS, FPS, DTF, PH, NPP, PYD, KY, HSW and SP (Table 3). The days to 75% flowering, %LLSI at 85 and 100 days after planting, PDY, KY, HSW, and SP were also significantly (*p* < 0.05) different due to the interaction effect between genotype and location. All the traits were significantly (*p* < 0.05) affected by the genotype x season interaction except number of pods per plant and rust score at 100 days after planting. Rust score at 85 days after planting did not show significant (*p* > 0.05) difference across seasons and locations. There was wide genotypic variation for most assessed traits (*p* < 0.001) due to genotype main effect for all traits except NPP and SP.

**Table 3:** Analysis of variance showing mean squares and significant tests for eight agronomic traits of 119 groundnut accessions evaluated across four environments (2 seasons × 2 locations)

Source of variation	DF	IPS	FPS	PH	DTF	NPP	%LLSI 85	%LLSI 100	%RI85	%RI100	PDY	KY	HSW	SP
Locations (L)	1	346.34***	97.38***	326.70***	75.76***	183.18***	251.39***	176.43***	0.07	34.57***	2222.70***	1589.26***	483.14***	24.65***
Rep	1	1.70*	6.30**	10.10**	0.56	0.61	39.54***	16.22***	6.27*	18.63***	1.01	0.40*	0.52	0.18
Block	7	2.33*	1.92	0.67	1.21	0.98	3.01**	2.52*	1.78	2.73**	0.48	0.63	2.51	1.33
Genotypes (G)	119	2.08***	2.68***	2.68***	1.71***	1.09	3.43***	4.66***	2.34***	4.00***	4.25***	3.17***	2.01***	1.04
Seasons (S)	1	101.81***	553.12***	312.41***	55.60***	1089.90***	584.17***	476.99***	1.42	5.67*	37.68***	31.98***	500.32***	14.91***
GxL	119	0.91	1.2	1.06	1.27*	0.96	1.33*	1.57***	1.01	0.90	4.58***	3.78***	2.27***	1.30*
GxS	119	1.29*	1.97***	1.31*	1.30*	0.80	1.32*	1.35*	1.34*	1.07	4.43***	3.68***	0.80	1.27*
SxL	1	899.66***	479.86***	2659.56***	43.37***	508.89***	164.81***	669.82	25.19***	116.62***	460.79***	436.14***	251.12***	54.05***
GxLxS	119	0.69	0.77	1.04	1.12	0.78	1.02***	1.71***	1.02	0.93	4.45***	3.26***	0.77	1.04
Residual		184.1	106.3	12.52	12.38	18.18	70.19	113	88.67	187.3	39,529	14,165	67.91	345.8

*DF* degrees of freedom, *IPS* initial plant stand, *FPS* final plant stand, *PH* plant height, *DTF* days to flowering, *NPP* number of pods per plant, %*LLSI 85* Percentage late leaf spot infection at 85 days after planting, %*LLSI 100* percentage late leaf spot infection at 100 days after planting, %*RI 85* percentage rust infection at 85 days after planting, %*RI* 100 percentage rust score infection at 100 days after planting, *PDY* pod yield, *KY* kernel yield, *HSW* hundred seed weight, *SP* shelling percent

*,** and *** represent significant differences at 0.05, 0.01 and 0.001 probability levels, respectively

The top 10 accessions with high pod yield and the five bottom performing genotypes are summarized in Table 4. These included ICGV-SM 16579 (967.5 kgha^−1^), ICGV-SM 16613 (926.8 kgha^−1^) and ICGV-SM 08587 (893.7 kgha^−1^) with moderate rust disease scores except for ICGV-SM 08587, which showed resistant to rust disease at hundred days after planting (Table 4). The mean pod yield across locations was 567.45 kgha^−1^ and kernel yield were 291 kgha^−1^. The highest average rust (35.17%) and late leaf spot (31.96%) scores were observed 100 days after planting compared to 85 days after planting. Pendo 98, which was used as a susceptible check showed moderate infection to both diseases (Supplementary Table 1) and it attained an average pod yield of 692.5kgha^−1^ The five bottom performing accessions in terms of pod yield were Narinut 15 (252.5 kgha^−1^), ICGV-SM 16574 (310.6 kgha^−1^), ICGV 95342 (318.1 kgha^−1^), ICGV-SM 08584 (338.4 kgha^−1^) and ICGV-SM 06711 (338.7 kgha^−1^). These accessions yielded below average pod yield. Narinut 15 and ICGV-SM 08584 showed resistance reaction to groundnut rust and late leaf spot.

**Table 4:** Mean values for agronomic traits of 119 groundnut accessions showing the top 10 and bottom 5 ranked genotypes based on mean pod yield (kg/ha) across four environments

Genotypes	IPS	FPS	PH	DTF	NPP	%LLSI85	%LLSI100	%RI85	%RI100	PDY	KY	HSW	SP
*Top 10 genotypes*													
ICGV-SM 16579	46.46	38.49	15.49	32.42	7.145	14.39	31.96	13.13	35.17	967.5	310.5	33.16	38.68
ICGV-SM 16613	46.53	34.43	12.99	31.28	10.86	7.87	18.88	13.12	27.68	926.8	432.8	34.7	42.57
ICGV-SM 08587	48.39	25.09	12.86	35.4	10.479	0.63	3.83	0.62	6.92	893.7	333.5	30.75	38.5
ICGV-SM 16555	41.95	32.13	14.65	34.14	6.054	12.53	17.02	7.51	19.33	869.4	433.8	26.2	49.05
ICGV-SM 16572	44.2	29.4	18.56	33.09	12.037	10.63	22.4	6.87	24.25	870.3	375.8	30.46	44.06
ICGV-SM 15546	40.91	31.15	13.8	33.04	9.775	5.01	7.04	3.12	8.31	844.7	426.7	28.54	46.59
ICGV 94,124	43.54	29.39	13.89	32.84	6.548	6.26	16.97	3.75	7.1	835.0	475.6	27.63	44.42
ICGV-SM 16,593	42.32	33.99	16.82	35.67	7.961	13.14	28.83	10.63	23.67	834.4	431.4	23.54	40.08
ICGV-SM 16589	41.73	38.96	17.21	31.92	7.289	15.63	26.46	13.75	29.4	810	431.7	24.33	43.74
ICGV-SM 15510	53.13	39.14	9.56	34	8.365	6.9	11.74	1.88	3.25	803.7	436.3	23.47	49.8
*Bottom 5 genotypes*													
Narinut 15	42.93	14.46	14.68	35.77	8.706	3.14	9.45	1.87	9.42	252.5	329.5	117.1	38.48
ICGV-SM 16,574	48.19	40.24	17.3	33.08	8.729	15.03	30.48	12.52	25.07	310.6	264	118.4	40.44
ICGV 95,342	40.28	31.64	16.26	34.06	9.953	13.13	22.7	16.25	23.39	318.1	475.6	163.3	48.46
ICGV-SM 08584	38.41	22.26	13.99	35.72	9.02	3.14	7.89	8.12	7.73	338.4	258.4	159.7	45.26
ICGV-SM 06711	31.27	20.01	15.34	34.34	10.79	7.52	14.14	6.89	22.73	338.7	282.3	165.6	39.71
Mean	40.28	31.2	16.10	33.6	8.69	10.49	18.75	10.14	21.75	567.45	291.16	27.86	39.96
LSD (5%)	19.69	15.61	7.48	3.68	7.07	11.79	16.94	9.68	14.51	341.9	190.2	11.50	16.22
CV %	49.81	51.27	47.23	11.17	82.96	114.09	91.96	97.24	67.92	61.49	66.48	42.02	34.82
R^2^	0.40	0.32	0.17	0.02	0.13	0.88	0.01	0.94	0.00	1	0.75	0.86	0.00
SED FIX	10.03	7.96	3.81	1.88	3.60	6.01	8.63	4.93	7.39	174.20	96.89	5.86	8.26

*IPS* = initial plant stand, *FPS* final plant stand, *PH* plant height, *DTF* days to flowering, *NPP* number of pods per plant, %*LLSI 85* Percentage late leaf spot infection at 85 days after planting, %*LLSI 100* percentage late leaf spot infection at 100 days after planting, %*RI 85* percentage rust infection at 85 days after planting, %*RI 100* percentage rust score infection at 100 days after planting, *PDY* pod yield, *KY* kernel yield, *HSW* hundred seed weight, *SP* shelling percent, *LSD* Least significant difference, *CV* coefficient of variation, R^2^ = coefficient of determination, *SED* Standard error of the mean differences

### Genotype × environment interaction effects on pod yield

**Fig. 1:**
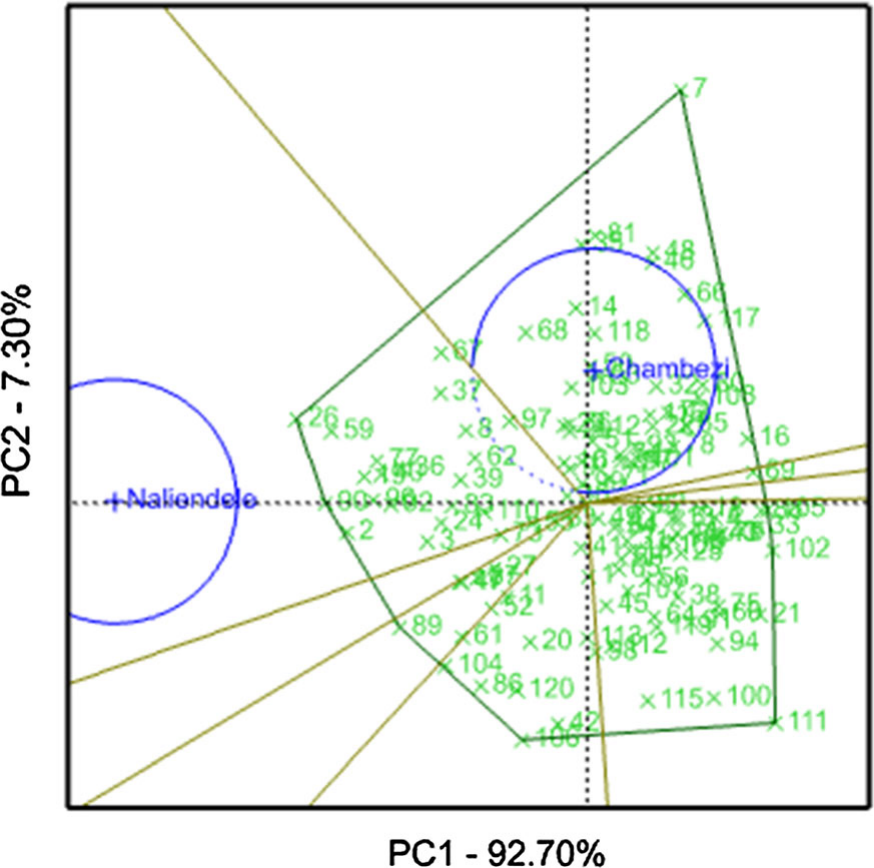
GGE-biplot showing the pod yield performance and stability of 119 accessions evaluated across two locations. Note: see codes of accessions in Table 1

**Fig. 2:**
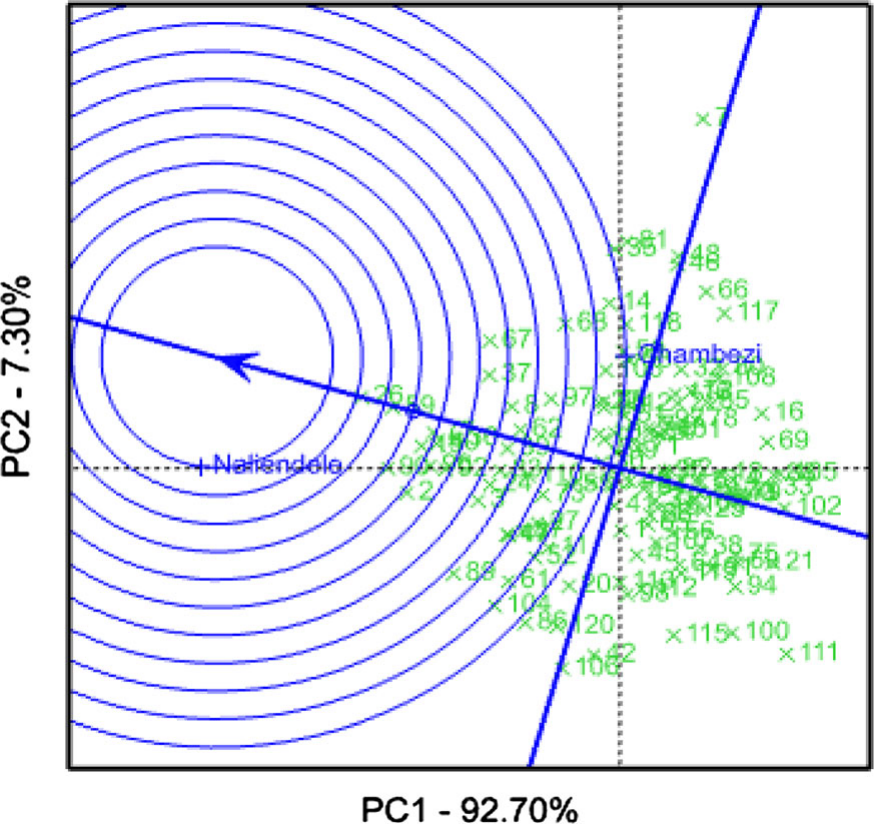
GGE bioplot comparing the test environments to the average environment coordinates based on pod yield of 119 accessions. Note: see codes of accesions in Table 1

The two axes in the GGE biplot accounted for 100% of the variation in the tested germplasm collections. Genotype ICGV-SM 16560, which represented with number 7 was found on the vertex of the polygon in the sector belonging to Chambezi site while ICGV-SM 16579, which represented with number 26 was the vertex genotype for TARI-Naliendele (Fig. 1). The two sites were distinctly different and did not belong to the same mega environment. Entries such as ICGV-SM 08584 (number 100), ICGV-SM 06737 (number 106) and Narinut 15 (number 111) did not show specific adaptation to a particular environment. TARI-Naliendele site had higher discriminatory capability and was more representative of the ideal environment compared to Chambezi (Fig. 2). In general, most genotypes exhibited lower mean performance at Chambezi site over both seasons compared to TARI-Naliendele. The average environment coordinate (AEC) view from the GGE analysis compares the mean performance of each genotype and its stability across the test environments. In this study, the AEC view showed genotype ICGV-SM 08587 (number 90) as the superior genotype and stable in terms of pod yield as located close to ideal genotype (Fig. 2).

### Correlations among traits

The Pearson correlation coefficients (r) among the traits were calculated and presented in Table 5. At TARI-Nalindele, the traits that exhibited significant correlation with KY were DTF (r = 0.133, *p* < 0.01) and NPP (r = 0.231, *p* < 0.01) (Table 5, above diagonal). Traits such as PH (r =  − 0.194, *p* < 0.01), %LLSI85 (r = -0.275, *p* < 0.01), %LLSI100 (r =  − 0.212, *p* < 0.01) and %RI100 (r =  − 0.204, *p* < 0.01) exhbited negative associations with KY. At Chambezi, KY was significantly correlated with FPS (r =  − 0.392), PH (r = 0.556), NPP (r = 0.637), %LLSI85 (r = − 0.153), %LLSI100 (r = 0.192), %RI100 (r = 0.358) and PDY (r = 0.639) at *p* < 0.01 (Table 5, below diagonal). The percentage LLS and rust infection were positively correlated in both test sites.

**Table 5:** Pearson's correlation coefficients showing the association of phenotypic traits of 119 groundnut accessions evaluated across two seasons at TARI-Naliendele (above diagonal) and Chambezi (below diagonal)

Traits	IPS	FPS	DTF	PH	NPP	%LLSI85	%LLSI100	%RI85	%RI100	PDY	KY	HSW	SP
IPS	–	0.54^**^	0.02	− 0.40^**^	0.22^**^	− 0.26^**^	− 0.32^**^	0.15^**^	− 0.07	− 0.12^**^	0.08	− 0.10^*^	− 0.14^**^
FPS	0.96^**^	–	− 0.29^**^	0.13^**^	− 0.05	0.16^**^	0.18^**^	0.07	0.04	0.07	0.01	0.08	0.12^**^
DTF	− 0.23^**^	− 0.26^**^	–	− 0.54^**^	0.33^**^	− 0.49^**^	− 0.56^**^	− 0.08	− 0.28^**^	− 0.28^**^	0.13^**^	− 0.11^*^	− 0.23^**^
PH	− 0.43^**^	− 0.41^**^	− 0.07	–	− 0.34^**^	0.66^**^	0.75^**^	− 0.06	0.25^**^	0.32^**^	− 0.20^**^	0.20^**^	0.37^**^
NPP	− 0.61^**^	− 0.59^**^	− 0.01	0.69^**^	–	− 0.45^**^	− 0.44^**^	− 0.06	− 0.24^**^	− 0.25^**^	0.23^**^	0.01	− 0.23^**^
%LLSI85	0.50^**^	0.52^**^	− 0.28^**^	− 0.08	− 0.24^**^	–	0.76^**^	− 0.04	0.37^**^	0.26^**^	− 0.26^**^	0.13^**^	0.25^**^
%LLSI100	0.24^**^	0.27^**^	− 0.24^**^	0.38^**^	0.17^**^	0.46^**^	–	− 0.09	0.37^**^	0.34^**^	− 0.21^**^	0.16^**^	0.33^**^
%RI85	0.31^**^	0.34^**^	− 0.22^**^	0.15^**^	0 − .01	0.46^**^	0.44^**^	–	0.36^**^	− 0.10^*^	− 0.07	− 0.14^**^	− 0.11^*^
%RI100	0.02	0.05	− 0.16^**^	0.47^**^	0.34^**^	0.18^**^	0.66^**^	0.42^**^	–	0.11^*^	− 0.20^**^	0.10^*^	0.11^*^
PDY	− 0.20^**^	− 0.16^**^	− 0.12^**^	0.46^**^	0.51^**^	0.02	0.27^**^	0.13^**^	0.35^**^	–	− 0.01	− 0.00	0.90^**^
KY	− 0.45^**^	− 0.39^**^	− 0.08	0.56^**^	0.64^**^	− 0.15^**^	0.19^**^	0.04	0.36^**^	0.64^**^	–	− 0.06	− 0.01
HSW	− 0.09	− 0.04	− 0.08	0.20^**^	0.23^**^	0.03	0.17^**^	0.08	0.21^**^	0.20^**^	0.39^**^	–	0.31^**^
SP	− 0.17^**^	− 0.13^**^	− 0.11^*^	0.45^**^	0.49^**^	0.03	0.31^**^	0.19^**^	0.38^**^	0.93^**^	0.65^**^	0.39^**^	–

*IPS* initial plant stand, *FPS* final plant stand, *PH* plant height, *DTF* days to flowering, *NPP* number of pods per plant, %*LLSI 85* Percentage late leaf spot infection at 85 days after planting, %*LLSI 100* percentage late leaf spot infection at 100 days after planting, %*RI 85* = percentage rust infection at 85 days after planting, %*RI 100* percentage rust score infection at 100 days after planting, *PDY* pod yield, *KY* kernel yield, *HSW* hundred seed weight, *SP* shelling percent

* and ** represent significant correlations at 0.05 and 0.01 probability levels, respectively

### Principal component analysis

The multi-variate relationship among traits was elaborated by the principal component analysis to show the contribution of each trait to the overall variation. Traits with high loadings on a given principal component (PC) are important as they account for more variation explained by that PC. The first four principal components accounted for 71.9% of the total variation (Table 6). The highest contributor to PC1 was Late leaf spot while the number of pods had the least PC1 contribution. For PC2, plant stand had the highest contribution followed by number of pods. Kernel yield and shelling percent had high contribution on PC3 while rust score had the highest leading on PC4. DAYS 75 had negative contribution on all components.

**Table 6:** Principal component scores and variance of each trait measured among 119 groundnut accessions across two seasons and two sites

Traits	PC1	PC2	PC3	PC4
IPS	− 0.071	0.905	0.1	0.171
FPS	0.14	0.887	− 0.009	0.186
DTF	− 0.505	− 0.264	− 0.165	− 0.18
PH	0.79	− 0.288	0.26	0.023
NPP	− 0.184	− 0.728	0.233	0.28
%LLSI85	0.836	0.253	0.003	0.023
%LLSI100	0.868	0.05	0.119	0.189
%RI85	0.063	0.21	− 0.038	0.782
%RI100	0.441	− 0.088	0.182	0.653
PDY	0.426	0.183	0.757	− 0.231
KY	− 0.03	− 0.23	0.8	0.158
HSW	0.032	− 0.076	0.575	0.332
SP	0.407	0.158	0.818	− 0.167
Eigenvalue	3.962	2.582	1.468	1.338
% of Variance	30.47	19.86	11.29	10.29
Cumulative %	30.474	50.333	61.622	71.911

*IPS* initial plant stand, *FPS* final plant stand, *DTF* days to flowering, *PH* plant height, *NPP* number of pods per plant, %*LLSI* Percentage late leaf spot infection at 85 days after planting, %*LLSI 100* percentage late leaf spot infection at 100 days after planting, %*RI 85* percentage rust infection at 85 days after planting, %*RI 100* percentage rust score infection at 100 days after planting, *PDY* pod yield, *KY* kernel yield, *HSW* hundred seed weight, *SP* shelling percent, *PC* principal component

### Genetic parameters of the SSR markers

In total, the 13 SSR markers used in this study amplified 38 alleles (Table 7). The number of alleles per marker ranged from 2 to 5 with a mean of 2.9 alleles per marker. The presence of allelic variants within the population was revealed by allele frequencies ranging from 0.319 to 0.992 with a mean of 0.713. Large variability was also observed among the markers for gene diversity, which ranged from 0.05 for m13_TE360 to a high of 1.56 for m13_PM035. The polymorphic information content values observed in this study ranged from 0.02 to 0.72 with a mean value of 0.34. Marker m13_TE360 showed the lowest PIC value of 0.02. The results also showed that only three of the markers used had PIC values ≥ 0.5. These were m13_PM035 (with PIC value of 0.72), m13_PGPseq_16C6 (0.66) and m13_PGPseq_10D4 (0.51).

**Table 7:** Genetic diversity estimates in 119 genotypes by using 13 SSR markers

Marker	Allele number	Allele frequency	Gene diversity	PIC
m13_GM2301	2	0.748	0.626	0.32
m13_IPAHM103	2	0.739	0.674	0.34
m13_PGPseq_10D4	3	0.630	1.031	0.51
m13_PGPseq_12F7	3	0.571	0.935	0.46
m13_PGPseq_13A10	3	0.513	0.857	0.43
m13_PGPseq_16C6	5	0.437	1.432	0.66
m13_PGPseq_17F6	3	0.807	0.679	0.31
m13_PGPseq_8E12	2	0.639	0.784	0.40
m13_PM035	5	0.319	1.557	0.72
m13_PM179	3	0.987	0.134	0.03
m13_SSR_HO115759	2	0.941	0.259	0.11
m13_TE360	2	0.992	0.049	0.02
m13_TE498	3	0.941	0.271	0.11
Mean	2.9	0.713	0.626	0.34

### Population structure

**Fig. 3:**
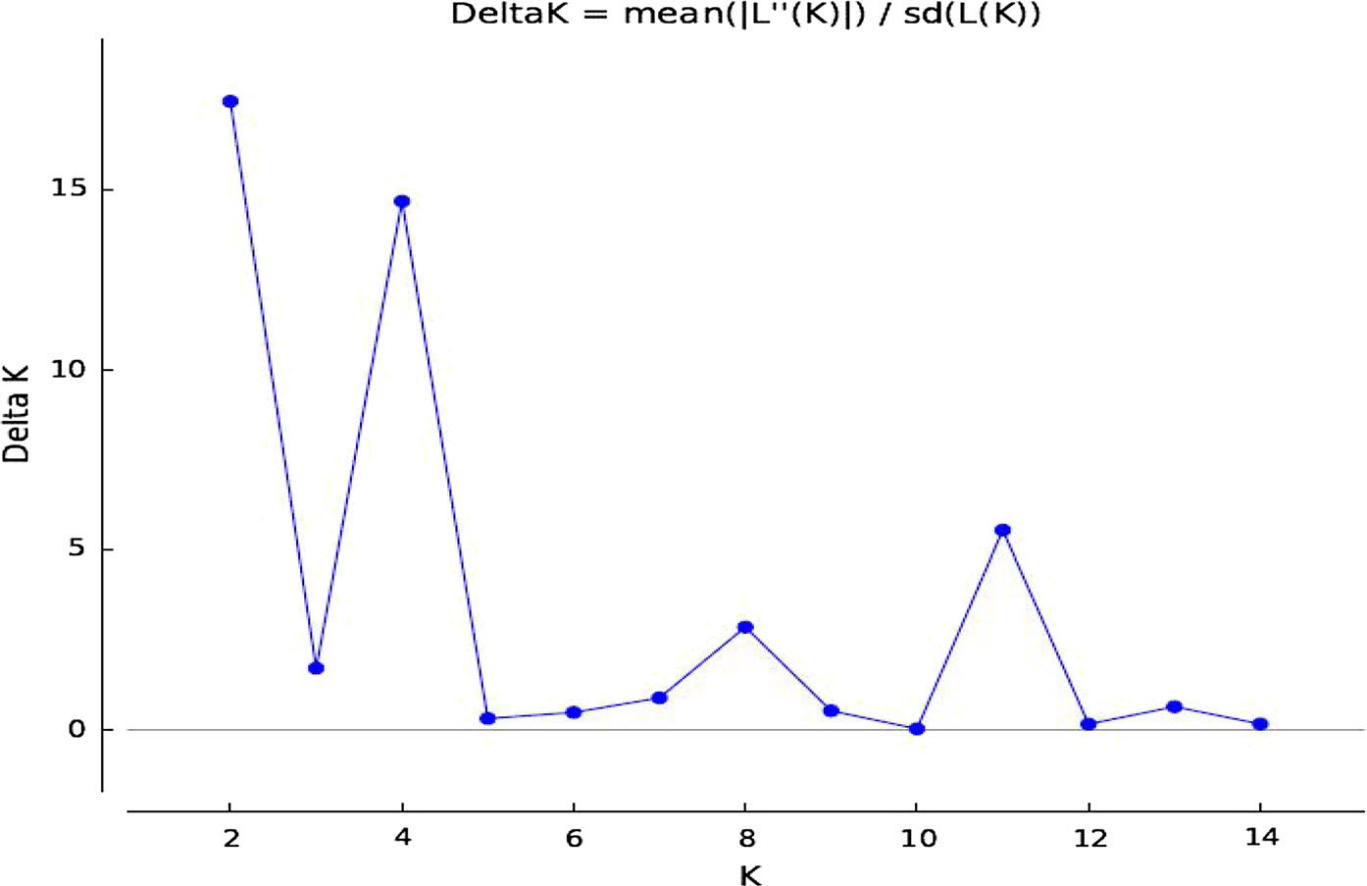
The best Delta K value for population structure among 119 groundnut genotypes

**Fig. 4:**
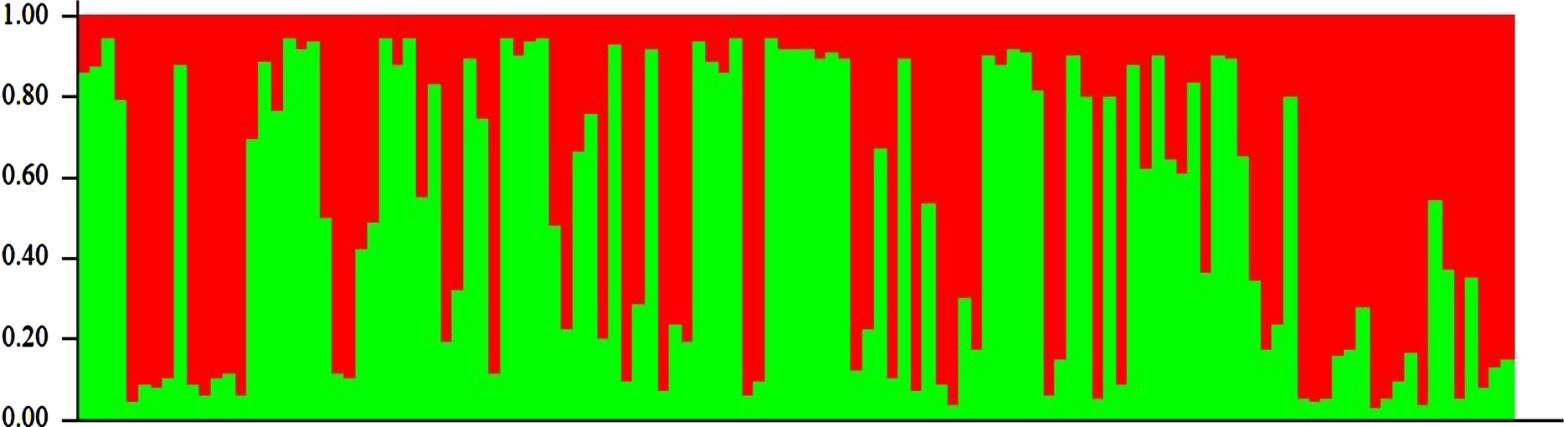
Estimated population structure of 119 groundnut genotypes with 13 SSR markers for K = 2 (Red = cluster 1, Green = cluster 2

The Evanno method estimated the best ‘K’ value to be 2 and, thus, the genotypes could be divided into two subpopulations (Fig. 3). The population structure analysis revealed that 74% of the accessions could be stratified into two sub-populations, while 26% could be regarded as admixtures. The two sub-populations were similar in size with sub-population 1 consisting of 36% of the genotypes while subpopulation 2 contained 37% (Fig. 4). Results showed that both sub-populations comprised of genotypes collected from different sources although most of the released genotypes were grouped in subpopulation 1 except Mangaka 09, which was grouped in subpopulation 2.

The expected heterozygosity in subpopulation 1 was 0.40 while for subpopulation 2 it was estimated to be 0.22 (Table 8). Allele frequency divergence between the two subpopulations was found to be 0.07. The level of genetic differentiation among the subpopulations was measured by estimating the fixation index (F_ST_). The results showed that sub population 2 with an F_ST_ of 0.47 was more differentiated than subpopulation 1, which had an F_ST_ of 0.01 (Table 8).

**Table 8:** Genetic clusters and their member genotypes, proportion of membership, expected heterozygosity and the mean fixation indices for 119 groundnut accessions

Cluster	Genotypes	(%) Membership	Expected heterozygosity	Fixation index (F_ST_)	Allele frequency divergence
1	ICGV-SM 08586, ICGV-SM 06718, ICGV-SM 15554, ICGV-SM 15559, ICGV-SM 16557, ICGV-SM 05570, ICGV-SM 16612, ICGV-SM 16617, CGV-SM 15534, CG 7, ICGV-SM 16565, ICGV-SM 15548 ICGV-SM 16559, Ndulima, ICGV-SM 15536, Nachingwea 09, ICGV-SM 05611, ICGV-SM 15510, ICGV-SM 15556, Narinut 15, ICGV-SM 16571, ICGV-SM 15524, ICG 12725, ICGV-SM 15546, ICGV 94114, ICGV-SM 15562, ICGV-SM 08587, ICGV-SM 15514, ICGV 95342, ICGV-SM 15529, ICGV-SM 06737, ICGV-SM 16558, ICGV-SM 08578, Masasi 09, ICGV-SM 16615, ICGV-SM 15538, ICGV-SM 16587, Kanyomwa, Naliendele 09, ICGV-SM 15567, ICGV-SM 08584,ICGV-SM 16597, ICGV-SM 16567	36	0.40	0.01	–
2	ICGV-SM 16567,ICGV-SM 1672, ICGV-SM 15558, ICGV-SM 16608, ICGV-SM 16601, ICGV-SM 16610, ICGV-SM 16586, ICGV-SM 16609, ICGV-SM 16556, ICGV-SM 16563, ICGV-SM 16595, ICGV-SM 16580, ICGV-SM 05569, ICGV-SM 16593, ICGV-SM 16603, ICGV-SM 16602, Mangaka 09, ICGV-SM 16579, ICGV 10879, ICGV-SM 16611, Local Tandahimba, ICGV-SM 16576, Mamboleo, ICGV-SM 16574,, ICGV-SM 16582, ICGV-SM 16598, ICGV-SM 16606, ICGV-SM 16591, ICGV-SM 16577, ICGV-SM 16568, ICGV-SM 16562, ICGV-SM 16578, ICGV-SM 16566, ICGV-SM 16583, ICGV-SM 16605, ICGV-SM 15542, ICGV-SM 06711, ICGV-SM 16600, ICGV-SM 16560, ICGV-SM 16588, Local Dodoma, ICGV-SM 16,604, ICGV-SM 16585, ICGV-SM 16581,ICGV-SM 16599. ICGV-SM 16592	38	0.22	0.47	0.07
Admixture	ICGV-SM 15531, ICGV-SM 16569, ICGV-SM 16570, ICGV-SM 16555, ICGV-SM 05616, ICGV-SM 15537, ICGV-SM 1684, ICGV-SM 16554, ICGV 93542, ICGV-SM 16561, ICGV 94114, ICGV-SM 87157, ICGV-SM 16564, ICGV-SM 16618, ICGV-SM 16594, ICGV-SM 15557, ICGV-SM 90704, ICGV-SM 16607, ICGV-SM 08581, ICGV-SM 06735, ICGV-SM 16575, ICGV-SM 16589, PENDO, ICGV-SM 15564, ICGV-SM 16616, ICGV-SM 16619, ICGV-SM 16590, CGV-SM 01514, ICGV-SM 16573, ICGV-SM 16613, ICGV-SM 16614	26	–	–	–

### Cluster analysis

**Fig. 5:**
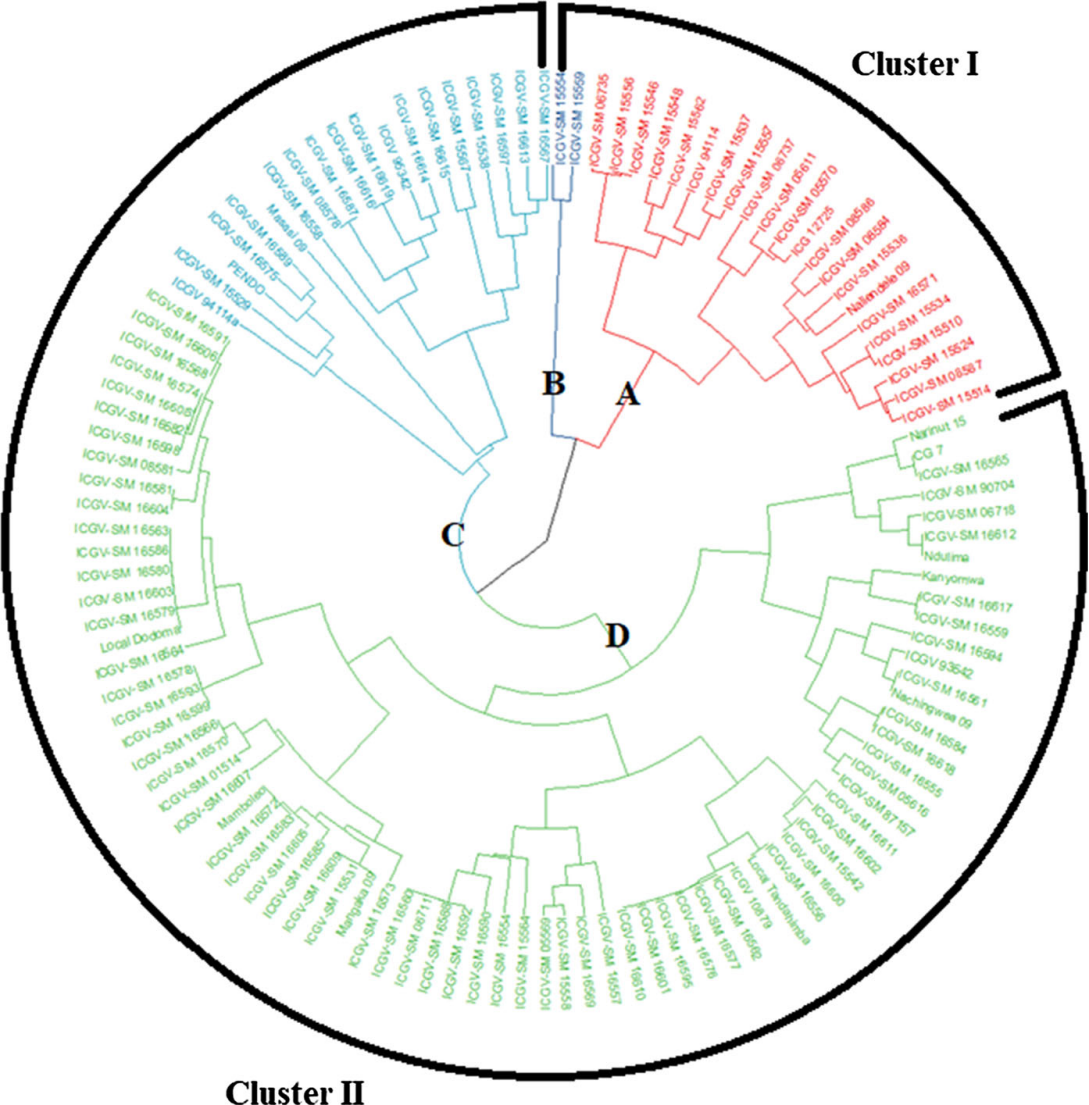
Neighbor joining hierarchical clustering of 119 groundnut accessions based on 13 SSR markers

The accessions were allocated into two main clusters (Fig. 5). Each cluster was further divided into two sub-clusters. Most individuals that were grouped in a cluster and its sub-cluster shared one or both parents showing close relatedness. Landraces were grouped in sub-cluster D within cluster 2 together with some lines from ICRISAT and released varieties. Five accessions (ICG 12725, ICGV-SM 06737, ICGV- SM 05570, ICGV-SM 15524 and ICGV-SM 15559), which were high yielding, but showed susceptibility to rust in the screening trial, and identified as potential parents for breeding were grouped into sub-cluster A. Sub-cluster C contained genotypes identified as high yielding and grouped together with Pendo 98, which is a popular cultivar in Tanzania and susceptible to rust. Landraces Kanyomwa and Narinut 15, which showed low yield but resistance to rust were grouped together in sub-cluster D. The analysis of molecular variance (AMOVA) among the 119 accessions estimated that 88% of the variation was due to intra-population variation while 2% was due to inter-population variation. There was also significant variation within accessions, which accounted for 10% of the variation (Table 9).

**Table 9:** Analysis of molecular variance (AMOVA) showing variation between and within the 119 groundnut accessions of different origin

Source	df	SS	MS	Est. Var	(%) Variation	*P* value
Between populations	1	14.499	14.499	0.065	2	0.160
Among individuals	117	803.833	6.870	3.252	88	0.001
Within individuals	119	43.500	0.366	0.366	10	0.031
Total	237	861.832	–	3.683	100	–

## Discussion

### Genotypic variation and mean performance

This study evaluated genetic variation among 119 accessions of groundnut using phenotypic traits and SSR markers as a preliminary step to identify suitable parental lines for rust resistance breeding.

The 119 accessions showed significant (*p* < 0.05) variation for yield and yield components showing that the germplasm could potentially provide vital genetic resources for groundnut improvement in Tanzania. The variation exhibited by phenotypic traits signify differences in genetic composition of the individuals (Liao 2014). The genotypes were sourced from different geographical locations where they could have adapted to local conditions and involved in localised natural selection, which could lead to genetic differentiation over time. Accessions such as ICGV-SM 16579, ICGV-SM 16613 and ICGV-SM 16555 from ICRISAT-Malawi had higher pod yield (PDY) compared to NARINUT 15, ICGV-SM 16598 and ICGV-SM 16557 that were acquired from TARI-Naliendele and ICRISAT-Malawi. The accessions from Malawi had comparable yields to the Tanzanian accessions, which could point to localized adaptation since Malawi and Tanzania share almost similar climatic factors and agricultural practices. Landraces and varieties adapted to different localities reflect differences in the climatic factors and agronomic practices in the environments where they were collected (Ren et al. 2014). The accessions showed significant variation for rust scores and performed differently in different sites, which will facilitate selection for resistant or tolerant lines for breeding and help to identify the best site for rust disease screening. Accessions such as ICGV-SM 06737, NARINUT 15 and Kanyomwa that scored low values for rust could be possible sources of genes for rust tolerance. Although these lines did not show comparable yield advantage, they can be used in crosses to introgress the resistance genes into genotypes with a high yield potential genetic background. Genotype ICGV-SM 16579 was identified as the best in terms of pod yield and stability while genotype ICGV-SM 08587 was more stable in terms of pod yield across the test environments. These accessions showed high level of rust disease susceptibility across the test environments, and therefore would not be selected as parental lines for rust resistance breeding but can provide the high yield potential genetic background. 16589.

### Trait associations

The relationships among yield components and disease response scores are critical in devising a selection strategy since selection of one trait may amplify or negatively affect performance in the other traits. The principal component (PC) analysis highlighted that late leaf spot, kernel yield, plant height, shelling percent and pod yield were mostly associated with PC1, showing that these traits accounted for much of the variation among the genotypes and could be used as the basis for selection. Accessions with higher performance in these traits could be selected for groundnut improvement. Rust scores were associated with PC4 as there was no wide range of variation for rust reaction among the accessions. This showed that most genotypes were more inclined towards susceptibility rather than resistance. Similarly, (Denwar et al. 2019) found that trait contribution to different PCs differed depending on the extent of variation for the particular trait among test genotypes. Pod yield, kernel yield and, late leaf spot, rust scored, and shelling percent are important yield components that can be used for indirect selection for yield due to their significantly correlation with yield. The correlations found in this study were in concurrence with Denwar et al. (2019), who also found that disease ratings were negatively correlated with yield while selection for number of pods and seeds per pod increased grain yield in soybean. The positive correlation between rust and late leaf spot shown in this study were confirmed in the previous reports (Narasimhulu et al*.* 2012; Narasimhulu et al. 2013). These diseases often occur together (Subrahmanyam et al., 1985; Branch and Culbreath, 2013) and accessions with resistance to these diseases are generally late maturing (Khedikar et al. 2010). The results also showed that there existed a highly negative correlation between rust scores and the number of pods per plant, which could be attributed to the decimation of foliage resulting in low photosynthetic capacity of the plant to accumulate a high number of pods. Leaf diseases are known to reduce yield through interfering with chloroplast integrity and causing abscission of leaves (Singh et al. 2011).

### Genetic diversity estimates based on the SSR markers

SSR markers are often preferred for genetic diversity study due to their co-dominance, simplicity, high polymorphism, repeatability, abundance, multi-allelic nature and their transferability within the genus *Arachis* (Moretzsohn et al. 2005; Pandey et al. 2012; Wang et al. 2012). The PIC ranges from 0.02 to 0.72 for the 13 SSR markers used in this study showed that the genotypes were genetically diverse, and the markers were able to discriminate the genotypes. Genetic variability emanates from differences in the genetic constitution of individuals, thus the panel included both closely related and divergent genotypes. It also shows that the markers used were efficient in discriminating the genotypes, which is fundamental in genetic studies to evaluate the extent of genetic variation in the gene pool. The highest PIC obtained in this study was comparably higher than 0.52 and 0.62 obtained by Varma et al. (2005) and Mace et al*.* (2006), respectively. Differences in PIC values are concomitant with differences in the markers and genotypes used in the studies. Nonetheless, it shows that the germplasm investigated in each of the studies exhibited adequate genetic variation that can be exploited during groundnut improvement. The variation is important for breeding for *Puccinia* resistance as it avails genotypes with diverse response to the pathogen and some of the genotypes could harbour resistance genes. The gene diversity obtained in this study (0.93), which is significantly higher than 0.11 and 0.59 obtained by Ren et al. (2014) and Wang et al. (2011), respectively, showed that there were many variants of the genes in this population because it included diverse genotypes that included released varieties, advanced lines and landraces. The high gene diversity also implies that the SSR markers used were highly polymorphic. Mace et al*.* (2006) asserted that the use of high polymorphic markers increases the potential of identifying high levels of gene diversity among test genotypes. A total of 38 alleles were revealed across the 13 polymorphic SSR loci in the 119 groundnut genotypes with an average of three alleles per locus, which was similar to four alleles per locus reported by Ren et al. (2014). There are a few markers that revealed five alleles per locus and were comparable to findings by Mace et al*.* (2006), who reported an average of six alleles per locus. This suggests that there is favourable allelic diversity, which is essential for assessment of genetic diversity. The variability in the number of alleles detected per locus by different reports might be due to the use of diverse genotypes.

### Population structure and clustering

The population structure, principal component and hierarchical clustering analyses were able to delineate the 119 accessions into two major clusters (Figs. 3 and 4). The optimal number of clusters in the population structure was based on the Evanno method (Earl and VonHoldt 2012), which has been widely used to confirm number of clusters in populations of different crops including cereals and legumes (Van Inghelandt et al. 2010; Ren et al. 2014; Denwar et al. 2019). The two identified clusters grouped the released varieties separately from the landraces while genotypes with similar genetic background were correctly placed in closely linked cluster and sub-clusters. Eighty-eight accessions were grouped into the two clusters while 31 accessions were admixtures. Admixtures could be regarded as separate clusters from the two main ones. The ability to delineate the germplasm is a significant step towards groundnut improvement in Tanzania as these genotypes form part of germplasm collection intended for use in country wide breeding programs. However, the low number of clusters could be a sign of narrow genetic diversity between populations. A narrow genetic base of groundnut had been reported by different authors (Mace et al*.* 2006; Mondal et al*.* 2008; Varshney et al. 2010). The narrow genetic variation could be a result of origin since all cultivated groundnuts originated in South America, through a limited number of interspecific hybridization and polyploidization (Pasupulet et al. 2013). Therefore, a wider range of accessions should be introduced to improve the current population for future breeding programs.

The mean fixation index (F_ST_) of 0.47 within subpopulation 2 indicates a higher genetic diversity within this subpopulation from which parental lines could be selected to produce variable populations for selection. The high F_ST_ was similar to 0.47 reported by (Wang et al. 2011). In contrast, the low F_ST_ found among genotypes in subpopulation 1, which was dominated by the crosses of JL 24, ICGV 94114, ICGV 95342 and ICGV 93437 lines from ICRISAT, could be a bottleneck for groundnut improvement by inter-crossing individuals within this subpopulation. Crosses between individuals in subpopulations 1 and 2 would be recommended to increase genetic variation and enhance genetic gain through active selection.

The first cluster consisted mainly of crosses of JL 24 and ICGV 94114, ICGV 90103 and ICGV 92092, ICGV 93437 and ICGV 95342, showing that the analysis managed to identify and group genetically related individuals (Table 8). The second cluster consists of C and D sub-groups of 19 and 76 genotypes, respectively. The D sub-group consisted of more genotypes compared to all subgroups. Ren et al. (2014) grouped 196 accessions of groundnut in 5 groups for both cluster and structure analyses. Most of the genotypes used in this study showed resistance to rust and LLS diseases except three genotypes (ICGV-SM 16585, ICGV-SM 16587 and ICGV-SM 16575), which showed comparable susceptibility to the susceptible check (Pendo 98).

The results showed that differences among individual accessions accounted for 88% of the variation, which means that the variation was less influenced by sources of collection or population structure. The remainder of the total variation was found among the populations, which could have been contributed by adaptation to different environments and the number of markers, which showed polymorphisms to groundnut rust. This agreed with Ren et al. (2014) who showed that only differences in geographic origin contributed less to the differentiation in groundnut collections from China. The variation within individuals could be attributed to factors such as low frequency mutations that induce localised genetic changes since groundnut is highly self-pollinating. Random mutations occur in nature and have been reported to be contributors to variation observed in most self-pollinating species (Sigurbjörnsson 1971; Oladosu et al. 2016).

## Conclusion

The accessions exhibited significant phenotypic variation in yield and yield component traits, which were underpinned by the genetic diversity. The trait associations revealed significant correlation between rust and late leaf spot severity and number of pods per plant providing a means for direct selection to improve yield and disease resistance. The SSR markers used in this study were able to deduce genetic variation among groundnut genotypes. The largest proportion of variation was attributed to individual differences, which is essential for improving rust resistance by crossing individuals from divergent clusters. The germplasm was stratified into two sub-populations despite being sourced from diverse collection sources showing that sources of collection were less important. Accessions ICGV-SM 15557, ICGV-SM 15559, ICGV-SM 06737, PENDO, ICGV-SM 16601, ICGV-SM 16589, ICGV-SM 05570, Kanyomwa, Narinut 15, ICG 12725, ICGV-SM 15524 and ICGV-SM 15567 exhibited low scores for rust resistance. Accessions ICGV-SM 16601, ICGV-SM 16589 had high mean performance for pod yield and were clustered in different clusters, which provides opportunity for their selection as divergent parental lines in groundnut breeding for enhanced yield. Furthermore, the current study identified accessions ICGV-SM 06737, ICGV-SM 16575, ICG 12725 and ICGV-SM 16608 of high diversity genotypically and in rust diseases could be used for development of rust mapping population, which will be useful resource for groundnut improvement.

## Electronic supplementary material

Below is the link to the electronic supplementary material.

Supplementary file1 (DOCX 22 kb)
