# Supplementary material for: Genetic diversity and population structure of groundnut (Arachis hypogaea L.) accessions using phenotypic traits and SSR markers: implications for rust resistance breeding
Source: Genet Resour Crop Evol. 2020 Sep 5;68(2):581–604. doi: 10.1007/s10722-020-01007-1 (PMC7811514; doi:10.1007/s10722-020-01007-1)
Supplement: Supplementary file 1 — Supplementary file1 (DOCX 22 kb) [file 10722_2020_1007_MOESM1_ESM.docx]

**Supplemental Table 1 Rust and late leaf spot disease reaction (%) of 119 groundnut accessions assessed in the study.**

| **S. No.** | **Accession (name/designation)** | **Rust reaction** | **Late leaf spot reaction** |
| --- | --- | --- | --- |
| 1 | ICGV-SM 16554 | 13.12 | 16.88 |
| 2 | ICGV-SM 16555 | 10.00 | 23.75 |
| 3 | ICGV-SM 16556 | 21.87 | 21.88 |
| 4 | ICGV-SM 16557 | 15.00 | 20.63 |
| 5 | ICGV-SM 16558 | 6.87 | 10.62 |
| 6 | ICGV-SM 16559 | 15.00 | 18.75 |
| 7 | ICGV-SM 16560 | 20.62 | 25.00 |
| 8 | ICGV-SM 16561 | 13.12 | 19.75 |
| 9 | ICGV-SM 16562 | 18.75 | 21.25 |
| 10 | ICGV-SM 16563 | 31.25 | 33.75 |
| 11 | ICGV-SM 16564 | 20.00 | 35.62 |
| 12 | ICGV-SM 16565 | 11.87 | 31.25 |
| 13 | ICGV-SM 16566 | 15.00 | 25.63 |
| 14 | ICGV-SM 16567 | 20.00 | 31.88 |
| 15 | ICGV-SM 16568 | 14.37 | 21.88 |
| 16 | ICGV-SM 16569 | 21.25 | 22.50 |
| 17 | ICGV-SM 16570 | 20.63 | 25.00 |
| 18 | ICGV-SM 16571 | 15.00 | 13.13 |
| 19 | ICGV-SM 16572 | 14.37 | 26.88 |
| 20 | ICGV-SM 16573 | 21.87 | 40.63 |
| 21 | ICGV-SM 16574 | 10.62 | 35.00 |
| 22 | ICGV-SM 16575 | 34.38 | 33.13 |
| 23 | ICGV-SM 16576 | 16.25 | 22.88 |
| 24 | ICGV-SM 16577 | 23.12 | 19.38 |
| 25 | ICGV-SM 16578 | 22.50 | 23.13 |
| 26 | ICGV-SM 16579 | 19.37 | 33.13 |
| 27 | ICGV-SM 16580 | 15.00 | 37.50 |
| 28 | ICGV-SM 16581 | 17.50 | 31.25 |
| 29 | ICGV-SM 16582 | 13.12 | 36.25 |
| 30 | ICGV-SM 16583 | 28.12 | 33.75 |
| 31 | ICGV-SM 16584 | 18.75 | 27.50 |
| 32 | ICGV-SM 16585 | 30.62 | 33.75 |
| 33 | ICGV-SM 16586 | 8.75 | 25.63 |
| 34 | ICGV-SM 16587 | 26.87 | 33.75 |
| 35 | ICGV-SM 16588 | 21.25 | 21.25 |
| 36 | ICGV-SM 16589 | 20.63 | 27.50 |
| 37 | ICGV-SM 16590 | 25.87 | 25.87 |
| 38 | ICGV-SM 16591 | 11.25 | 11.25 |
| 39 | ICGV-SM 16592 | 31.25 | 31.25 |
| 40 | ICGV-SM 16593 | 13.50 | 13.50 |
| 41 | ICGV-SM 16594 | 13.75 | 23.75 |
| 42 | ICGV-SM 16595 | 23.75 | 35.00 |
| 43 | ICGV-SM 16597 | 15.00 | 19.38 |
| 44 | ICGV-SM 16598 | 18.75 | 26.25 |
| 45 | ICGV-SM 16599 | 18.33 | 27.92 |
| 46 | ICGV-SM 16600 | 23.12 | 43.75 |
| 47 | ICGV-SM 16601 | 19.37 | 17.88 |
| 48 | ICGV-SM 16602 | 18.75 | 28.13 |
| 49 | ICGV-SM 16603 | 20.62 | 22.88 |
| 50 | ICGV-SM 16604 | 20.00 | 28.75 |
| 51 | ICGV-SM 16605 | 25.63 | 37.50 |
| 52 | ICGV-SM 16606 | 13.13 | 31.25 |
| 53 | ICGV-SM 16607 | 13.75 | 28.75 |
| 54 | ICGV-SM 16608 | 28.75 | 28.75 |
| 55 | ICGV-SM 16609 | 20.00 | 25.25 |
| 56 | ICGV-SM 16610 | 17.50 | 18.13 |
| 57 | ICGV-SM 16611 | 6.87 | 18.13 |
| 58 | ICGV-SM 16612 | 10.00 | 6.88 |
| 59 | ICGV-SM 16613 | 18.12 | 18.50 |
| 60 | ICGV-SM 16614 | 20.00 | 20.38 |
| 61 | ICGV-SM 16615 | 8.75 | 10.62 |
| 62 | ICGV-SM 16616 | 19.38 | 18.13 |
| 63 | ICGV-SM 16617 | 13.13 | 26.88 |
| 64 | ICGV-SM 16618 | 16.25 | 21.88 |
| 65 | ICGV-SM 16619 | 26.25 | 34.38 |
| 66 | ICGV 93542 | 23.13 | 24.38 |
| 67 | ICGV-SM 15510 | 5.00 | 18.75 |
| 68 | ICGV-SM 15514 | 9.38 | 10.00 |
| 69 | ICGV-SM 15524 | 5.63 | 10.00 |
| 70 | ICGV-SM 15529 | 24.38 | 15.63 |
| 71 | ICGV-SM 15531 | 12.87 | 15.38 |
| 72 | ICGV-SM 15534 | 10.00 | 16.25 |
| 73 | ICGV-SM 15536 | 17.50 | 15.63 |
| 74 | ICGV-SM 15537 | 10.62 | 18.50 |
| 75 | ICGV-SM 15538 | 13.12 | 18.75 |
| 76 | ICGV-SM 15542 | 33.19 | 26.88 |
| 77 | ICGV-SM 15546 | 3.75 | 10.00 |
| 78 | ICGV-SM 15548 | 17.92 | 15.83 |
| 79 | ICGV-SM 15554 | 8.13 | 16.25 |
| 80 | ICGV-SM 15556 | 6.87 | 20.63 |
| 81 | ICGV-SM 15557 | 4.37 | 18.13 |
| 82 | ICGV-SM 15558 | 23.75 | 8.75 |
| 83 | ICGV-SM 15559 | 6.25 | 17.50 |
| 84 | ICGV-SM 15562 | 8.75 | 21.88 |
| 85 | ICGV-SM 15564 | 16.25 | 15.00 |
| 86 | ICGV-SM 15567 | 2.50 | 10.00 |
| 87 | ICGV-SM 90704 | 15.00 | 7.88 |
| 88 | ICGV 94114 | 16.87 | 33.13 |
| 89 | ICGV-SM 08578 | 8.13 | 15.00 |
| 90 | ICGV-SM 08587 | 8.75 | 4.38 |
| 91 | ICGV-SM 08586 | 8.12 | 7.88 |
| 92 | CG 7 | 6.87 | 10.88 |
| 93 | ICGV-SM 08581 | 10.62 | 9.75 |
| 94 | ICG 12725 | 3.12 | 10.00 |
| 95 | ICGV-SM 05570 | 3.13 | 3.13 |
| 96 | ICGV 94124 | 6.25 | 18.13 |
| 97 | ICGV-SM 06718 | 8.75 | 8.75 |
| 98 | ICGV-SM 05611 | 18.12 | 15.63 |
| 99 | ICGV-SM 05569 | 8.12 | 9.38 |
| 100 | ICGV-SM 08584 | 8.12 | 8.75 |
| 101 | ICGV-SM 06735 | 10.62 | 18.13 |
| 102 | ICGV 95342 | 13.75 | 29.38 |
| 103 | ICGV-SM 05616 | 4.38 | 15.63 |
| 104 | ICGV-SM 87157 | 10.62 | 18.13 |
| 105 | ICGV-SM 06711 | 16.87 | 18.13 |
| 106 | ICGV-SM 06737 | 1.25 | 9.38 |
| 107 | ICG 10879 | 7.50 | 15.63 |
| 108 | ICGV-SM 01514 | 6.87 | 22.50 |
| 109 | Masasi 09 | 5.62 | 8.75 |
| 110 | Pendo 98 | 15.00 | 15.00 |
| 111 | Narinut 15 | 1.87 | 9.38 |
| 112 | Mangaka 09 | 15.00 | 10.00 |
| 113 | Naliendele 09 | 16.87 | 19.38 |
| 114 | Nachingwea 09 | 6.88 | 17.00 |
| 115 | Kanyomwa | 3.75 | 6.88 |
| 116 | Local Dodoma | 18.12 | 31.25 |
| 117 | Mamboleo | 22.50 | 26.25 |
| 118 | Local Tandahimba | 22.50 | 25.63 |
| 119 | Ndulima | 17.50 | 25.00 |
